# DO OPPOSITES ATTRACT OR AGGRAVATE? THE IMPACT OF INTERGENERATIONAL SOLIDARITY ON WELL-BEING

**DOI:** 10.1093/geroni/igac059.2342

**Published:** 2022-12-20

**Authors:** Rachel Scott, Acacia Lopez, Marin Olson, Danielle Nadorff

**Affiliations:** Mississippi State University, Starkville, Mississippi, United States; Mississippi State University, Starkville, Mississippi, United States; Mississippi State University, Mississippi State, Mississippi, United States; Mississippi State University, Mississippi State, Mississippi, United States

## Abstract

Family Systems Theory states that values are transmitted between generations within families and, while many of these values are similar between immediate generations, there may be more differentiation in values between generation gaps. Ideological differences between generations may potentially cause subsequent tension and fluctuations in well-being. The current study sought to examine the moderating effect of ideological differences (religious and political) on the relation between how emotionally close grandparents perceive themselves being with younger generations and grandparental well-being. Participants included 419 grandparents (age: M = 76, SD = 5.33), 716 adult children (age: M = 53, SD = 4.16), and 638 adult grandchildren (age: M = 29, SD = 5.52) from the 8th wave of the Longitudinal Study of Generations (LSOG) data set. Well-being in grandparents was positively correlated with perceived emotional closeness among both of the intergenerational dyads. Religious ideological differences between grandparents and grandchildren were found to negatively influence grandparental well-being; this relation was also found to be significantly different from political ideological differences within the same intergenerational dyad. While moderation was not achieved for either dyad, the overall model fit was found to be excellent, suggesting its utility for further research into the relation between the study variables. These findings indicate that there is a complex relation between perceived emotional closeness, ideological beliefs, and the well-being of grandparents that warrant additional attention within the literature and may inform interventions related to intergenerational communication and well-being. Further implications for these findings will be discussed.

